# Aerosolized nicotine from e-cigarettes alters gene expression, increases lung protein permeability, and impairs viral clearance in murine influenza infection

**DOI:** 10.3389/fimmu.2023.1076772

**Published:** 2023-03-14

**Authors:** Mazharul Maishan, Aartik Sarma, Lauren F. Chun, Saharai Caldera, Xiaohui Fang, Jason Abbott, Stephanie A. Christenson, Charles R. Langelier, Carolyn S. Calfee, Jeffrey E. Gotts, Michael A. Matthay

**Affiliations:** ^1^ Cardiovascular Research Institute, University of California, San Francisco, San Francisco, CA, United States; ^2^ Division of Pulmonary, Critical Care, Allergy and Sleep Medicine, University of California, San Francisco, San Francisco, CA, United States; ^3^ Department of Medicine, University of California, San Francisco, San Francisco, CA, United States; ^4^ Chan Zuckerberg Biohub, San Francisco, CA, United States; ^5^ Division of Infectious Diseases, University of California, San Francisco, San Francisco, CA, United States; ^6^ Department of Anesthesia, University of California, San Francisco, San Francisco, CA, United States

**Keywords:** influenza, e-cigarette (e-cig), ARDS, nicotine, viral load

## Abstract

E-cigarette use has rapidly increased as an alternative means of nicotine delivery by heated aerosolization. Recent studies demonstrate nicotine-containing e-cigarette aerosols can have immunosuppressive and pro-inflammatory effects, but it remains unclear how e-cigarettes and the constituents of e-liquids may impact acute lung injury and the development of acute respiratory distress syndrome caused by viral pneumonia. Therefore, in these studies, mice were exposed one hour per day over nine consecutive days to aerosol generated by the clinically-relevant tank-style Aspire Nautilus aerosolizing e-liquid containing a mixture of vegetable glycerin and propylene glycol (VG/PG) with or without nicotine. Exposure to the nicotine-containing aerosol resulted in clinically-relevant levels of plasma cotinine, a nicotine-derived metabolite, and an increase in the pro-inflammatory cytokines IL-17A, CXCL1, and MCP-1 in the distal airspaces. Following the e-cigarette exposure, mice were intranasally inoculated with influenza A virus (H1N1 PR8 strain). Exposure to aerosols generated from VG/PG with and without nicotine caused greater influenza-induced production in the distal airspaces of the pro-inflammatory cytokines IFN-γ, TNFα, IL-1β, IL-6, IL-17A, and MCP-1 at 7 days post inoculation (dpi). Compared to the aerosolized carrier VG/PG, in mice exposed to aerosolized nicotine there was a significantly lower amount of Mucin 5 subtype AC (MUC5AC) in the distal airspaces and significantly higher lung permeability to protein and viral load in lungs at 7 dpi with influenza. Additionally, nicotine caused relative downregulation of genes associated with ciliary function and fluid clearance and an increased expression of pro-inflammatory pathways at 7 dpi. These results show that (1) the e-liquid carrier VG/PG increases the pro-inflammatory immune responses to viral pneumonia and that (2) nicotine in an e-cigarette aerosol alters the transcriptomic response to pathogens, blunts host defense mechanisms, increases lung barrier permeability, and reduces viral clearance during influenza infection. In conclusion, acute exposure to aerosolized nicotine can impair clearance of viral infection and exacerbate lung injury, findings that have implications for the regulation of e-cigarette products.

## Introduction

Acute respiratory distress syndrome (ARDS) has a substantial health burden, causing 200,000 deaths annually in the United States and significant morbidity in survivors (1). Bacterial and viral pneumonia are the most common cause of ARDS (2), with COVID-19 increasing the incidence of ARDS by ten-fold in the United States (3). Prior to the COVID-19 pandemic, influenza was the most common virus responsible for pneumonia and ARDS (4), and new influenza viral strains continue to present a risk for potential future pandemics (5). While viral respiratory infections are a major risk factor for ARDS, not all viral infections lead to ARDS and environmental factors have an important role in developing disease (6).

The course of influenza infections is modified by cigarette smoke, which is associated with increased influenza severity in case-control (7) and cohort (8) studies and higher influenza mortality in large population studies (9). Additionally, animal studies have demonstrated that cigarette smoke exposure increases lung injury and mortality from influenza infection (10). Moreover, primary upper respiratory epithelial cells from smokers have impaired antiviral immune responses, dysregulated cytokine release, and greater viral shedding compared to non-smokers (11). These studies establish cigarette smoke as a significant risk factor for worse outcomes from influenza and highlight the urgent public health need to understand how the chemicals in cigarette smoke modify the host response to viral infections.

Nicotine is an addictive stimulant found in cigarette smoke that can also be inhaled *via* electronic nicotine delivery systems, or e-cigarettes. These devices use a heated metal coil to aerosolize nicotine solubilized in an e-liquid mixture of vegetable glycerin (VG) and propylene glycol (PG) as well as other additives like flavoring compounds (12, 13). Studies have shown that exposure to e-cigarette aerosols has injurious effects in the respiratory system linked to different chemicals in these products (14). However, there is still limited knowledge on the direct effects of nicotine on pulmonary function and pathophysiology as reports of cigarette smoking and e-cigarette use have largely not investigated nicotine in isolation but rather in a mixture with other chemicals. Prior studies found that intradermally administered nicotine blunted T cell and B cell responses in animals (15, 16), but the impact of aerosolized nicotine on acute respiratory viral infections remains poorly understood. With the rapid proliferation of e-cigarette use, studies are needed to determine how their specific chemical constituents impact lung injury and viral infections (17).

In this study, we tested three hypotheses. First, we hypothesized that exposure to e-cigarette aerosols generated from an e-liquid containing VG/PG only (i.e., the carrier) or an e-liquid containing VG/PG plus nicotine would increase pro-inflammatory responses in the distal airspaces of the lungs. Second, we hypothesized that following e-cigarette aerosol exposure, mice infected with influenza A (H1N1 PR8 strain) would have an impaired capacity to clear the influenza viral infection and show evidence of greater lung injury, as measured by increased lung protein permeability. Third, we hypothesized that prior exposure to e-cigarette aerosols would cause increased expression of pro-inflammatory transcriptomic pathways in the lungs of mice infected with influenza.

## Methods

### Animals and exposures to e-cigarette aerosols and cigarette smoke

**Figure 1:**
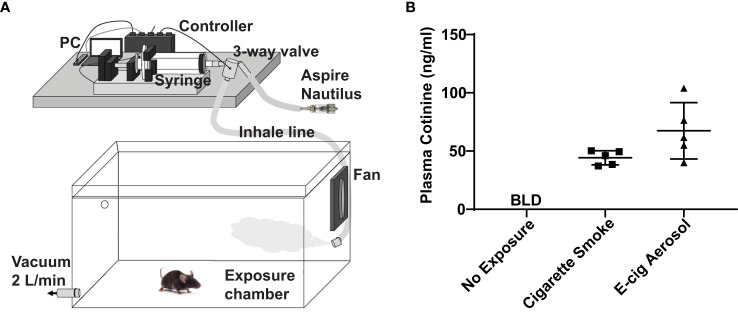
E-cigarette aerosol exposure system achieves appropriate plasma cotinine levels in mice. **(A)** Mice were housed in a plexiglass chamber. E-cigarette aerosol was generated by the tank-style Aspire Nautilus and drawn into a syringe by the Gram Universal Vaping Machine controlled by programmed computer software. The aerosol was injected through a three-way valve into the exposure chamber and circulated by a fan. A vacuum was used to maintain a steady-state and draw in fresh air and aerosol. **(B)** Plasma cotinine levels in mice exposed for nine days, one hour/day, to aerosol generated by the Nautilus device containing an e-liquid mixture of vegetable glycerin (VG), propylene glycol (PG), and 36 mg/ml nicotine was not significantly different (P = 0.099, unpaired t-test) to that of mice exposed to cigarette smoke. Cigarette smoke exposures were over five hours (single day) with a Teague TE-10 smoking machine using 3R4F Kentucky research cigarettes, as in our prior studies (18, 19). Plasma cotinine in unexposed mice was below the limit of detection (BLD). Data are presented in nanogram/milliliter (ng/ml). Each data point represents one mouse.

Adult (8–12 weeks old) C57BL/6 mice were purchased from the National Cancer Institute, housed in pathogen-free housing, and cared for in accordance with NIH guidelines by the Laboratory Animal Resource Center of the University of California, San Francisco (UCSF). All experiments were conducted under protocols approved by the UCSF Institutional Animal Care and Use Committee. For e-cigarette exposures, mice were housed in a plexiglass chamber that was filled with air (controls) or e-cigarette aerosol delivered by a Gram universal vaping machine *via* a syringe pump (**Figure 1A**), as we have done in prior studies (20, 21). The e-cigarette used in this study was the re-fillable tank-style Aspire Nautilus atomizer (4 volts, 1.8-ohm coil, 9 watts) filled with an e-liquid mixture of a 1/1 ratio of vegetable glycerin and propylene glycol (VG/PG) with or without free base nicotine. Inhale (syringe draw) and exhale (syringe infusion) periods were set at 4.1 and 2.3 seconds with a puff volume of 80 ml. 10 puffs were initially injected into the chamber over approximately 1 minute, followed by 110 puffs over 1 hour. To create steady-state conditions, the chamber was evacuated to an external fume hood at a constant rate of 2.0 L/min during the exposure, using a calibrated flowmeter (Dwyer) to draw in a mixture of fresh aerosol and room air. Cigarette smoke exposures were done over five hours with 100 mg/m^3^ total suspended particles (TSP) using a Teague TE-10 smoking machine with 3R4F Kentucky research cigarettes, as we have done in prior studies (18, 19).

### Bronchoalveolar lavage and measurements of protein, cell counts, inflammatory cytokines, and mucin protein

Mice were anesthetized with isoflurane followed by overdose with ketamine. After tracheal cannulation, bronchoalveolar lavage (BAL) was performed with two 250 μL aliquots of PBS and subsequently combined together. The total number of cells in the BAL were counted using a Cytosmart automated cell counter (Corning). Total protein in the cell-free BAL was measured with the Pierce BCA Assay (Thermo Fisher Scientific). Inflammatory cytokines in the BAL were measured using a Luminex assay with a ProcartaPlex multiplex kit (Thermo Fisher Scientific) for the following analytes: eotaxin, granulocyte-macrophage colony stimulating factor (GM-CSF), CXC motif chemokine ligand 1 (CXCL1), interferon (IFN) -γ, interleukin (IL) -1β, IL-10, IL-12p70, IL-13, IL-17A, IL-18, IL-2, IL-22, IL-23, IL-27, IL-4, IL-5, IL-6, IL-9, IFN-inducible protein (IP) -10, monocyte chemoattractant protein (MCP) -1, MCP-3, macrophage inflammatory protein (MIP) -1α, MIP-1β, MIP-2, regulated upon activation, normal T-cell expressed and secreted (RANTES), and tumor necrosis factor α (TNFα). Mucin 5 subtype AC (MUC5AC) in the BAL was measured by ELISA (Novus Biologicals).

### Influenza virus infections

Mice were anesthetized with isoflurane, and 300 foci-forming units of influenza A/H1N1/Puerto Rico/8/34 (PR8) dissolved in 30 µl of PBS was administered nasally, as in our prior work (22). Mice were monitored daily for morbidity and mortality as per institutional animal welfare guidelines.

### Influenza viral load measurements

Mice not used for BAL were overdosed with ketamine and underwent bilateral thoracotomy. Blood was collected by right ventricular puncture to obtain plasma for cotinine measurements. The left lung was placed in RNA Shield (Zymo Research), incubated at 4°C overnight, and then frozen at −20°C. Frozen lungs were thawed, minced, homogenized, and then samples extracted using a Zymo Quick viral RNA kit (Zymo Research). RNA presence and quality in extracts was tested using a DS-11 Fx+ spectrophotometer (DeNovix), and cDNA was created using a High Capacity cDNA Reverse Transcription Kit (Applied Biosystems). RT-PCR was performed using a SYBR Green Kit (Bio-Rad) and a LightCycler (Roche) with primers specific for influenza viral nucleoprotein A, VNP, (forward: CAGCCTAATCAGACCAAATG, backward: TACCTGCTTCTCAGTTCAAG) and murine GAPDH (forward: AAGGTCATCCCAGAGCTGAA, backward: CTGCTTCACCACCTTCTTGA), as in our prior work (23). The mRNA concentration specific for VNP to that of GAPDH was calculated. To quantify the number of viral particles, the right lung was homogenized in 1 ml of PBS and the homogenate was plated onto 96-well plates of confluent MDCK cells. 1 hour later, samples were decanted and replaced with serum-free media containing tosyl phenylalanine chloromethyl ketone trypsin at 1.5 μg/ml. After 15 hours, the MDCK cells were fixed in 100% methanol and then underwent indirect immunocytochemistry using mouse anti-influenza A (MAB 8257, Millipore) at 1.25 μg/ml, followed by biotinylated horse anti-mouse (Vector Laboratories), and the biotin/avidin system (PK-4002, Vector Laboratories) with diaminobenzidine as a chromogen. Samples were quantified in triplicate over 10^5^ dilutions; foci were counted in wells containing 30-100 discrete foci.

### RNA sequencing and analysis

RNA-seq libraries were prepared using the NEB Ultra-II kit and underwent paired-end sequencing on an Illumina NovaSeq 6000. Sequences were aligned to the murine genome using STAR (24). Sample quality metrics, including total protein coding reads, number of non-zero reads, hierarchical clustering, and principal component analysis were reviewed prior to downstream analyses. Differential expression analysis was performed using *DESeq2* using default filters (25). Empirical Bayesian posterior log-fold changes were calculated using *apeglm*. Differentially expressed genes were identified using independent hypothesis weighted false discovery rates (FDR) (26). Genes were ranked using their shrunken log_2_ fold changes for Gene Set Enrichment Analysis (GSEA). GSEA was performed using the C5 Gene Ontology pathway reference from *msigdbr*, which maps human gene symbols from MSigDB to murine gene symbols. Raw sequencing data are available under NCBI BioProject ID PRJNA891788.

### Statistical analyses

Comparisons between groups were made with ANOVA or unpaired t-test. Repeated measures ANOVA was used for comparisons of multiple groups over more than one time point, and two-way interaction terms were created for treatment group and time. P < 0.05 was considered to be statistically significant. Analyses were done using Prism 8 software (GraphPad). Data are presented as mean and standard deviation. Significant differentially expressed genes and GSEA pathways were identified by an adjusted p-value less than 0.1.

## Results

### Nine days of e-cigarette aerosol exposure achieved plasma cotinine levels comparable to cigarette smoke exposure

E-cigarette aerosol generated by an Aspire Nautilus device, containing a 1/1 mixture of VG/PG (i.e., the carrier) with 36 mg/ml of nicotine, was drawn by a Gram Universal Vaping machine controlled by programmed software, as illustrated in **Figure 1A**. The aerosol was then pushed through a three-way valve into a plexiglass chamber in which mice were housed for one hour per day over nine days. After the nine days of e-cigarette aerosol exposure, there was no significant difference (P = 0.099) in the plasma concentration of cotinine, the metabolite of nicotine produced by the liver (27), to that of mice that were exposed to cigarette smoke for five hours (**Figure 1B**). Subsequent experiments were performed with exposures to aerosols generated using an e-liquid of only the carrier VG/PG or of VG/PG containing 36 mg/ml nicotine (VG/PG/Nic).

### Exposure to e-cigarette aerosol containing nicotine elicited inflammatory cytokine production in the lung airspaces

**Figure 2:**
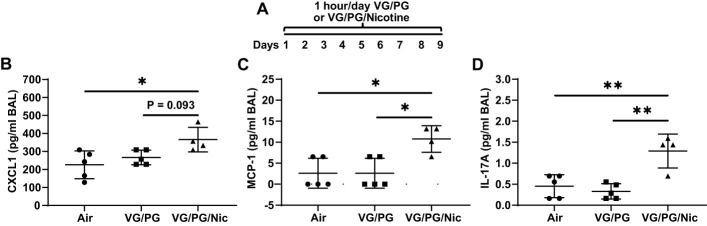
Nicotine-containing e-cigarette aerosol elicits an inflammatory response in the airspaces. **(A)** Timeline of e-cigarette exposures. **(B–D)** CXC motif chemokine ligand 1, CXCL1 **(B)**, monocyte chemoattractant protein-1, MCP-1 **(C)**, and interleukin-17A, IL-17A **(D)**, were significantly greater in the bronchoalveolar lavage (BAL) from mice exposed to nicotine-containing aerosol (VG/PG/Nic) than in mice exposed to only VG/PG (i.e., the carrier) or to air, suggesting a nicotine-specific effect. * P < 0.05, ** P < 0.01, 1-way ANOVA with Tukey’s multiple comparisons test. Data are presented in picogram/milliliter (pg/ml). Each data point represents one mouse.

Mice were exposed to air (control) or to aerosols generated from an e-liquid containing the carrier VG/PG only or VG/PG with 36 mg/ml nicotine (VG/PG/Nic), enabling isolation of nicotine-specific effects (**Figure 2A**). Following nine days of exposure (1 hour/day), bronchoalveolar lavage (BAL) was done. The concentrations of the inflammatory CXCL1, MCP-1, and IL-17A, **Figures 2B–D** respectively, were significantly higher in the BAL from mice exposed to aerosolized VG/PG/Nic than those exposed to the carrier VG/PG or to air. There was no significant difference in BAL cytokine concentrations between mice exposed to VG/PG only and those to air. There was no significant difference in the number of total cells counted in the BAL between Air (17 ± 9 cells/µl), VG/PG (11 ± 4 cells/µl), and VG/PG/Nic (16 ± 12 cells/µl). Additionally, the weight of mice was not significantly different between the Air (28.1 ± 1.9 g), VG/PG (27.5 ± 1.4 g), and VG/PG/Nic (28.1 ± 1.6 g) exposure groups.

### Nicotine-containing e-cigarette aerosol increased the lung protein permeability and blunted the increase in BAL MUC5AC concentration induced by influenza infection

**Figure 3:**
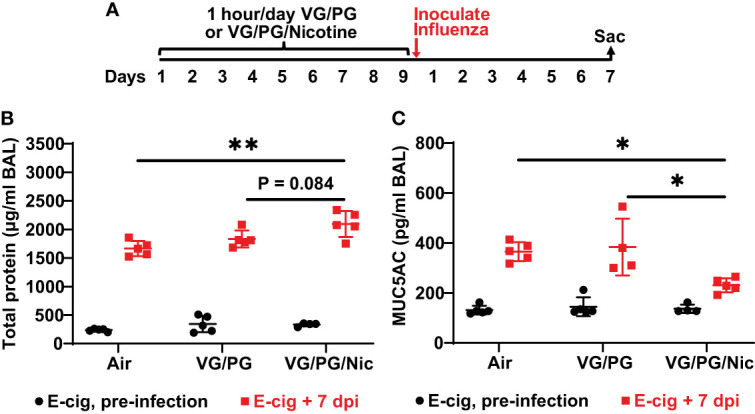
Aerosolized nicotine increases the lung barrier permeability to protein and blunts the increase of MUC5AC in the airspaces induced by influenza infection. **(A)** Timeline of the experiment. After nine days of e-cigarette aerosol exposure, mice were intranasally inoculated with 300 foci-forming units of influenza A H1N1 PR8 strain and then sacrificed 7 days post inoculation (dpi) to perform BAL. **(B)** Total protein concentration, presented in microgram/milliliter (µg/ml), in the BAL was not significantly different between groups after e-cigarette exposure before influenza infection. BAL protein significantly increased (p < 0.05) after influenza infection and was significantly higher at 7 dpi in the mice that were exposed to nicotine-containing aerosol (VG/PG/Nic) than those exposed to air. **(C)** Mucin 5 subtype AC (MUC5AC) concentration, presented in picogram/milliliter (pg/ml), in the BAL was not significantly different between groups after e-cigarette exposure before infection. BAL MUC5AC was significantly increased (p < 0.05) by influenza infection at 7 dpi but to a lesser degree in mice exposed to aerosolized VG/PG/Nic which was significantly lower compared to mice exposed to the aerosolized carrier VG/PG or to air. * P < 0.05, ** P < 0.01, 1-way ANOVA with Tukey’s multiple comparisons test between the three groups at 7 dpi. Each data point represents one mouse.

Total protein concentration measured in the BAL from mice after nine days of e-cigarette aerosol exposure was not significantly different between the three exposure groups (**Figure 3B**). After e-cigarette exposures, mice that were inoculated intranasally with influenza A (H1N1 PR8 strain) were sacrificed 7 days post inoculation (dpi) to perform BAL (**Figure 3A**), and BAL protein was significantly increased (p < 0.05) by influenza infection at 7 dpi (**Figure 3B**). BAL at 7 dpi from mice exposed to aerosolized VG/PG/Nic had significantly higher total protein concentration than those exposed to air and a trend for higher BAL total protein compared to mice exposed to the carrier VG/PG though this difference did not reach significance (P = 0.084). Mucin 5 subtype AC (MUC5AC) in the BAL was not significantly different after nine days of e-cigarette exposure compared to air (**Figure 3C**). Influenza infection significantly increased (p < 0.05) MUC5AC in the BAL at 7 dpi but to a lesser degree in mice exposed to VG/PG/Nic aerosol which had significantly lower BAL MUC5AC than mice exposed to the aerosolized carrier VG/PG or to air.

### E-cigarette aerosol exposure increased influenza-induced inflammatory cytokine production in mouse lung airspaces

**Figure 4:**
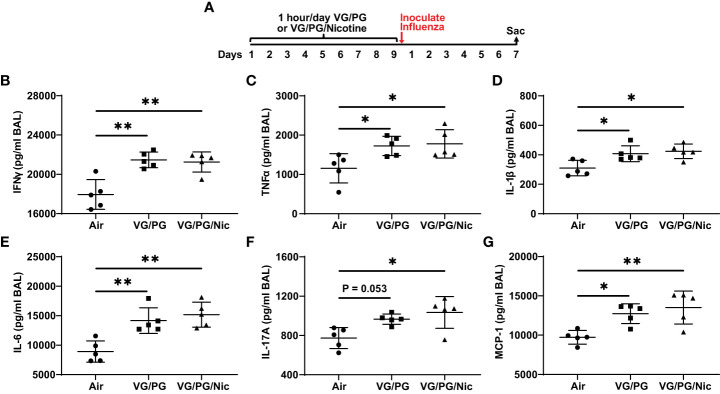
E-cigarette aerosol exposure increases influenza-induced production of key inflammatory cytokines in the airspaces 7 days after infection. **(A)** Timeline of the experiment. B-G: Infection with influenza resulted in BAL concentrations of interferon-γ, IFN-γ **(B)**, tumor necrosis factor α, TNFα **(C)**, interleukin-1β, IL-1β **(D)**, IL-6 **(E)**, IL-17A **(F)**, and monocyte chemoattractant protein-1, MCP-1 **(G)**, that were greater in mice exposed to e-cigarette aerosols with (VG/PG/Nic) or without (VG/PG) nicotine compared to control mice (air). This effect could be attributed to the VG/PG that serves as a carrier for other constituents of the e-liquid. * P < 0.05, ** P < 0.01, 1-way ANOVA with Tukey’s multiple comparisons test. Data are presented in picogram/milliliter (pg/ml). Each data point represents one mouse.

At 7 dpi with influenza, BAL from mice exposed to e-cigarette aerosol generated from the carrier VG/PG or from VG/PG/Nic had significantly higher levels of IFN-γ, TNFα, IL-1β, IL-6, IL-17A, and MCP-1 compared to mice exposed to air (**Figure 4**). No differences in cytokine expression between VG/PG and VG/PG/Nic groups were observed, suggesting that the increased production of cytokines in the airspaces induced by influenza infection was attributable to aerosols containing VG/PG and not an effect specific to nicotine. Additionally, there were non-significant trends for greater weight loss induced by influenza infection at 7 dpi in mice exposed to aerosolized VG/PG (16.4 ± 2.0 %; P = 0.2748) or VG/PG/Nic (17.3 ± 2.7 %; P = 0.1361) compared to Air (13.2 ± 4.3 %). There was no significant difference in the number of total cells counted in the BAL between Air (299 ± 46 cells/µl), VG/PG (286 ± 61 cells/µl), and VG/PG/Nic (255 ± 68 cells/µl).

### Nicotine-containing e-cigarette aerosol impaired influenza viral clearance in murine lungs

**Figure 5:**
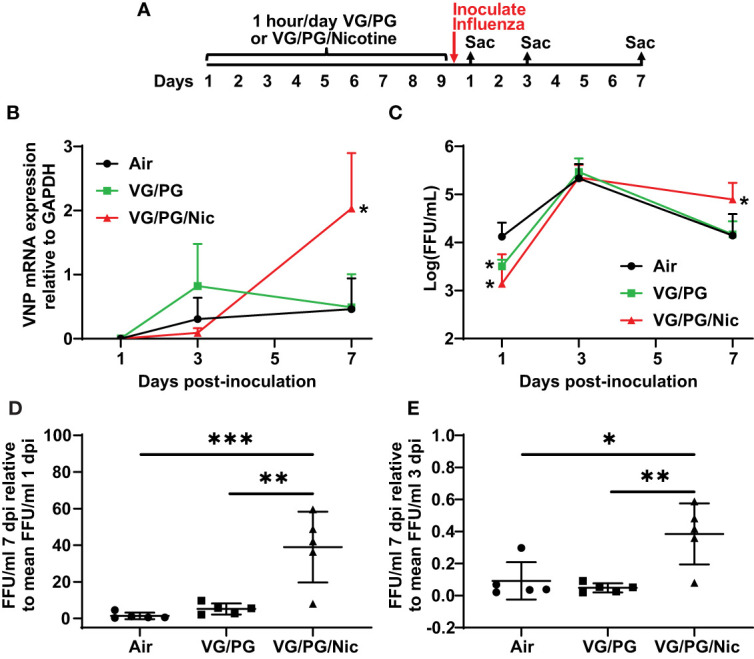
Nicotine-containing e-cigarette aerosol exposure impairs clearance of influenza virus. **(A)** Timeline of the experiment with separate groups of mice sacrificed 1-, 3-, and 7-days post-inoculation (dpi) with influenza. **(B)** Viral nucleoprotein (VNP) mRNA expression in the lung homogenate significantly increased (p < 0.05) from 3 dpi to 7 dpi in mice exposed to aerosolized nicotine (VG/PG/Nic) but not in those exposed to the aerosolized carrier VG/PG or to air. The presented data was normalized to GAPDH mRNA expression. * P < 0.05, mixed effects model with Tukey’s multiple comparisons test at each timepoint, comparing VNP/GAPDH expression between the three groups. **(C)** The concentration of viral particles, presented in foci-forming units/milliliter (FFU/ml), in the lung homogenate peaked at 3 dpi and decreased at 7 dpi in mice exposed to the aerosolized carrier VG/PG or to air but remained significantly elevated at 7 dpi in those exposed to aerosolized nicotine (VG/PG/Nic). * P < 0.05, mixed effects model with Tukey’s multiple comparisons test at each timepoint, comparing FFU/ml between the three groups. **(D, E)** The absolute FFU/ml counted at 7 dpi as a ratio to the mean FFU/ml at 1 dpi **(D)** and at 3 dpi **(E)**. * P < 0.05, ** P < 0.01, *** P < 0.001, 1-way ANOVA with Tukey’s multiple comparisons test. Each data point represents the mean and standard deviation from five mice in **(B, C)**, and represents one mouse in **(D, E)**.

After nine days of e-cigarette exposure and subsequent inoculation with influenza, mice were sacrificed at 1, 3 and 7 dpi (**Figure 5A**) to harvest the lungs that were then homogenized to quantify viral load. The expression of influenza viral nucleocapsid protein (VNP) was determined by qPCR and the number of infective viral particles (i.e., foci-forming units, FFUs) was also quantified. VNP mRNA expression was not significantly different at 1 dpi while at 7 dpi the lungs of mice previously exposed to aerosolized VG/PG/Nic had significantly higher VNP mRNA than those exposed to the aerosolized carrier VG/PG or to air (**Figure 5B**). Influenza virus FFUs, an assay of virions capable of producing an active infection, (**Figure 5C**) increased in all three groups from 1 dpi to 3 dpi and then decreased at 7 dpi but was significantly higher in the lungs of mice exposed to VG/PG/Nic than those exposed to VG/PG only or to air. Virus FFUs in the mice exposed to aerosolized VG/PG/Nic was approximately 40-fold higher at 7 dpi than at 1 dpi, whereas it returned to baseline in the mice exposed to the aerosolized carrier VG/PG or to air (**Figure 5D**). A similar trend was evident from comparing virus FFUs at 7 dpi to that of 3 dpi (**Figure 5E**), in which there was significantly less viral clearance in the mice that were exposed to VG/PG/Nic aerosol.

### Aerosolized nicotine caused transcriptomic changes consistent with increased pro-inflammatory signaling and lung injury following influenza infection

**Figure 6:**
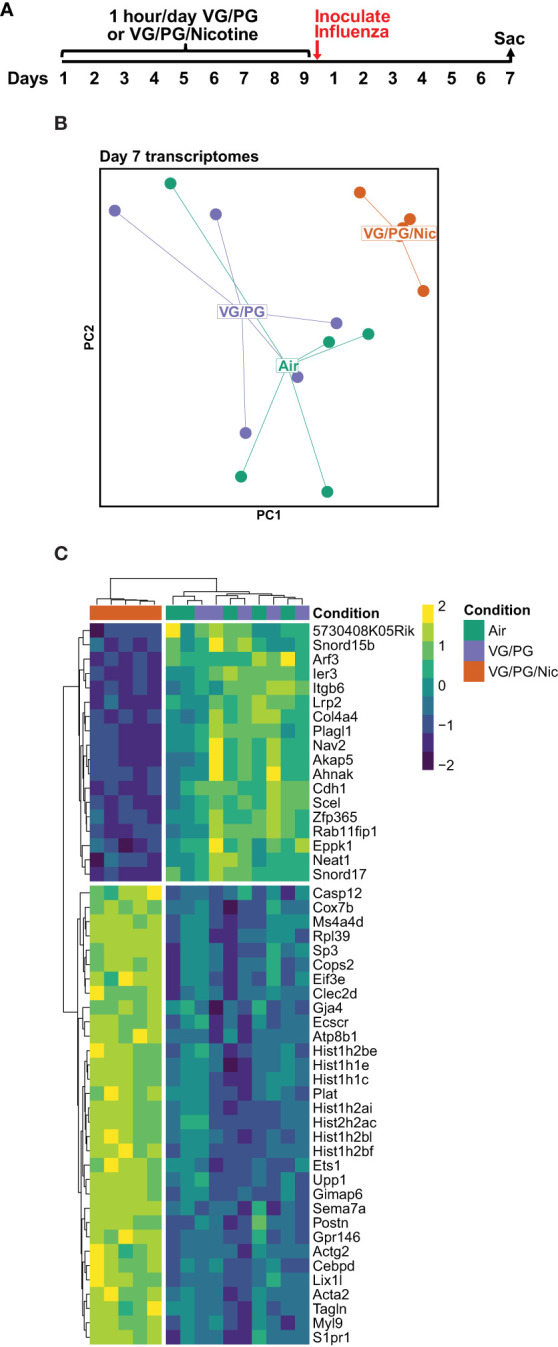
Pulmonary gene expression during influenza infection is shifted by prior nicotine exposure. **(A)** Timeline of the experiment. Seven days after influenza inoculation, mice were sacrificed to collect whole lungs for RNA extraction. **(B)** Principal components (PC1 and PC2) analysis of murine lung gene expression after 7 days of infection with influenza following exposures to e-cigarette aerosols containing nicotine (VG/PG/Nic), only the carrier (VG/PG), or to air. **(C)** Heatmap of the top 50 differentially expressed genes by adjusted *P* value in the lungs from influenza-infected mice exposed to aerosolized VG/PG/Nic compared to those exposed to VG/PG or to air. Each data point represents one mouse.

**Figure 7:**
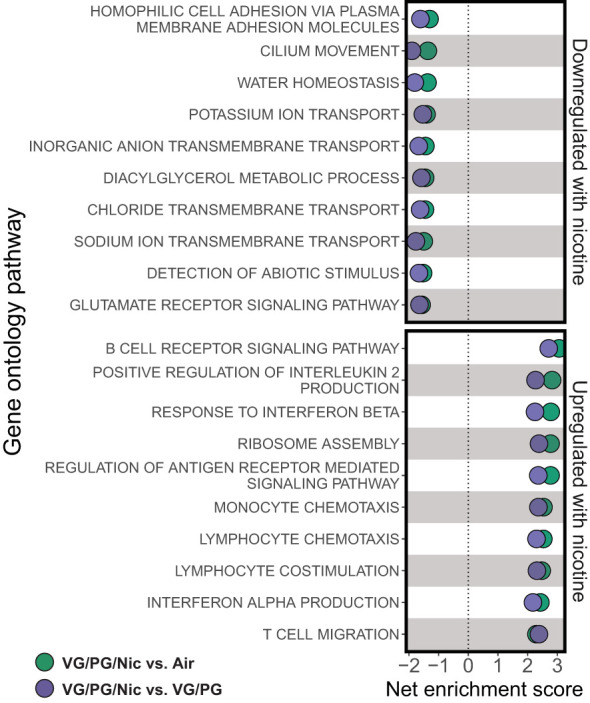
Aerosolized nicotine downregulates pathways for ciliary defense mechanisms and ion transport, and upregulates immune activation during influenza infection. Gene set enrichment analysis based on differentially expressed genes for the top downregulated and upregulated pathways in the lungs of influenza-infected mice exposed to nicotine-containing e-cigarette aerosol (VG/PG/Nic) compared to those exposed to nicotine-free aerosol (VG/PG, purple) or to air (green). Aerosolized nicotine exposure caused a downregulation of pathways associated with ciliary functions, ion transport, and water balance and an upregulation of innate and adaptive immune signaling seven days after influenza infection.

Lung gene expression in influenza-infected mice exposed to aerosolized VG/PG/Nic markedly differed from those exposed to the carrier VG/PG or to air (**Figure 6** and **Supplementary Data 1**) when measured at 7 dpi. Notably, there was no significant differential gene expression between the VG/PG and air exposure groups (**Supplementary Data 1**). Pathway analysis revealed exposure to VG/PG/Nic aerosol relatively downregulated genes involved in the function of cilia, ion transport, and fluid balance following influenza infection (**Figure 7** and **Supplementary Data 2**) compared to mice exposed to VG/PG or to air. The lungs of mice exposed to aerosolized VG/PG/Nic also had an upregulation of pathways related to both innate and adaptive immune signaling including the responses of macrophages, B cells, and T cells to infection (**Figure 7** and **Supplementary Data 2**) compared to control mice exposed to air and mice exposed to aerosol of the carrier VG/PG.

## Discussion

We investigated the effects of VG/PG and nicotine from e-cigarette aerosols during influenza infection in a well-studied murine model. Exposure to aerosolized VG/PG without nicotine increased influenza infection-induced pro-inflammatory cytokine production in the lung airspaces. E-cigarette aerosols containing nicotine increased pro-inflammatory pulmonary gene expression, blunted mucin production, decreased viral clearance and increased lung barrier permeability during influenza infection. We also found that aerosolized nicotine exposure induced pro-inflammatory cytokine production in the lung airspaces.

With our e-cigarette aerosol exposure system (**Figure 1A**), plasma levels of cotinine, the nicotine metabolite produced by the liver (27), in mice were comparable to those reported in smokers (28–30) and e-cigarette users (31, 32). Additionally, the plasma cotinine levels from exposures to nicotine-containing e-cigarette aerosols were not significantly different from those mice exposed to cigarette smoke. This finding is important as it demonstrated that our exposure system models real-world use. Furthermore, the 9-watt coil and the 36 mg/ml nicotine concentration in the e-liquid used in this study are in line with the trend for lower power and higher nicotine concentrations in popular e-cigarette products (33).

This relatively short-term exposure to nicotine modified inflammatory signaling in the airspaces prior to influenza exposure. Mice exposed one hour/day over nine days to e-cigarette aerosol containing nicotine had significantly higher airspace levels of CXCL1, MCP-1, and IL-17A compared to mice exposed to the carrier VG/PG and to control mice exposed to air. Given that CXCL1 is a potent neutrophil chemoattractant (34–36), MCP-1 is involved in leukocyte trafficking and inflammatory responses (37, 38), and IL-17A is involved in host defenses (39), this suggests that aerosolized nicotine exposure may have primed the lungs of mice for a pro-inflammatory response at baseline before influenza infection. Interestingly, despite the higher levels of CXCL1, MCP-1, and IL-17A after aerosolized nicotine exposure, there was no evidence of acute lung injury as protein in the airspaces was not significantly higher than in the lungs of mice exposed to VG/PG or to air. This finding is consistent with previous reports of inflammatory responses caused by nicotine-containing e-cigarette aerosols without tissue injury in murine lungs (40, 41), and the profile of protein expression in the lung airspaces is altered significantly more by the presence of nicotine than by VG/PG alone (42). Additionally, there is evidence of inflammation in the lungs of e-cigarette users inhaling nicotine (43–45). However, there is limited knowledge about the direct effects of nicotine in the lungs as most reports have studied cigarette smoke or a combination of different chemicals found in e-cigarette products on the market. The findings from this study, designed to isolate the effects of nicotine and the carrier fluid (VG/PG), help fill knowledge gaps for how aerosolized nicotine directly impacts lung inflammation with and without infection.

Our results suggest VG/PG and nicotine have significant and distinct effects on the biology of influenza infection. Influenza stimulates the production of several inflammatory cytokines as part of the host defense responses to the virus (46, 47). The H1N1 PR8 strain of influenza used in this study is mouse-adapted (48, 49) and known to cause viral pneumonia and acute lung injury in mice (22, 23). Compared to controls, mice exposed to the carrier VG/PG or to VG/PG/Nic both had higher levels of pro-inflammatory cytokines in BAL fluid seven days after influenza infection. The addition of nicotine did not lead to an increase in pro-inflammatory cytokine concentrations compared to the carrier alone, indicating this effect was attributable to the aerosolized e-liquid carrier VG/PG and not specific to nicotine, which is in line with a previous report (50). Exposure to aerosols containing VG and/or PG is known to cause inflammation and injury in the lungs (51). Several studies have reported that aerosolized VG/PG promotes pro-inflammatory responses in the airway epithelium (41, 52–54) and during infection (50, 55–58). This study adds to the evidence that aerosolized VG/PG itself can have potentially harmful effects in the form of heightened inflammatory responses during influenza infection, an important implication considering VG and PG are currently generally recognized as safe (59).

The 300 FFUs of influenza virus used in this study is a relatively moderate inoculum dose, compared to our prior experiments (22, 23); this dose causes inflammation and lung injury but allows for recovery without significant mortality. Both the mice that were exposed to the aerosolized carrier VG/PG and those to air had reduced influenza virus FFUs in their lungs at 7 dpi than at 3 dpi and returned to viral loads comparable to that at 1 dpi (**Figure 5**), suggesting the infection was under control at 7 dpi and recovery had initiated in these mice. In contrast, virus FFUs remained significantly higher at 7 dpi and VNP mRNA expression increased significantly from 3 dpi to 7 dpi in mice exposed to nicotine-containing aerosol, indicating a nicotine-specific impairment of viral clearance and failure to manage the infection. Nicotine-containing e-cigarette exposure downregulates innate immune system genes in alveolar epithelial cells (60), impairs macrophage phagocytic capacity (61–65), reduces dendritic cell functions (66–68), and blunts the oxidative burst of neutrophils (69), all of which are important for managing and clearing viral infections (70–72). Interestingly, nicotine administered intradermally was reported to impair leukocyte migration and influenza viral clearance in mice (73, 74).

In this study, we have identified that nicotine in e-cigarette aerosols blunts the increase in the BAL concentration of mucin protein MUC5AC induced by influenza infection. Mucins are key proteins that form the gel-like structure of mucus that lines the respiratory tract, which is a critical host defense mechanism against pathogens (75, 76). MUC5AC is a major mucin making up respiratory mucus (77) and its production in airway epithelial cells is increased by exposures to cigarette smoke (78, 79) and to e-cigarette aerosols (80, 81). Moreover, the increased production of MUC5AC is linked to aerosolized VG/PG and suppressed by the presence of nicotine (41, 53, 79, 82). E-cigarette aerosol exposure alone did not significantly change BAL MUC5AC concentration in this study. Influenza infection increased BAL MUC5AC, but the increase was significantly less in mice exposed to aerosolized nicotine than in mice exposed to the carrier VG/PG or to air. Influenza is known to induce the production of mucins in the respiratory tract as part of the host defense response (83), and increasing MUC5AC production is protective and improves viral clearance during influenza infection (84). Given this protective role, the reduced BAL MUC5AC concentration following influenza infection may partially explain the impaired viral clearance in the mice that were exposed to aerosolized nicotine.

While we did not identify significant differences between VG/PG and VG/PG/Nic exposure in a selected panel of BAL cytokines, an unbiased analysis of whole lung homogenate gene expression identified a marked difference in pulmonary gene expression caused by nicotine during influenza infection. Pathway analysis revealed that nicotine-containing aerosol exposure downregulated the expression of genes linked to the function of cilia, ion transport, and fluid balance in murine lungs infected with influenza compared to mice exposed to the aerosolized carrier VG/PG or to air. Cilia on the cells lining the respiratory tract are a critical early point of contact between pathogens and the host, enabling the clearance of pathogens trapped in mucus out of the airways (85, 86). Influenza, in turn, is known to reduce mucosal and ciliary functions and gene expression to evade this host defense mechanism (87). Additionally, e-cigarette exposure impairs ciliary beating (88) and suppresses gene expression in airway epithelial cells *in vitro* (89), in a murine model of COPD (40), and in e-cigarette users (53, 90) who develop reduced cough sensitivity (91). Moreover, the downregulation of ion transport genes with nicotine exposure also points to impaired ciliary function, which depend on a tightly regulated balance of sodium and chloride (92). Taken together, the finding of an association between exposure to aerosolized nicotine and downregulation of pathways linked to ciliary function during influenza infection may partially explain the higher viral load in the lungs from nicotine-exposed mice.

The impaired influenza virus clearance could have enabled the infection to persist and prolong viral shedding (93), which is associated with longer and more severe illness in hospitalized patients (94–97). Higher viral load has also been linked to mortality in influenza (98, 99) and to higher rates of transmissibility in the community (100, 101). Additionally, prolonged infection can cause lung injury as evidenced by increased barrier permeability and accumulation of proteinaceous fluid in the lung airspaces, which is quantifiable by measuring total protein in the BAL (102, 103). Though BAL protein was not increased by nine days of e-cigarette exposure, it was elevated by influenza infection and more so in mice with prior exposure to nicotine-containing e-cigarette aerosol, indicating the nicotine exacerbated lung injury caused by influenza. Moreover, nicotine downregulated pathways linked to fluid balance and ion transport during influenza infection, which are central functions of the alveolar epithelium for maintaining lung homeostasis (104). Though there was a trend for greater influenza-induced weight loss in nicotine-exposed mice and a weaker trend with VG/PG exposure, we have reported in our prior studies that there is poor correlation between body weight and lung injury in this model of PR8 influenza infection (22).

The higher influenza viral load caused by nicotine occurred in parallel with an upregulation of genes related to innate immune responses (e.g., type 1 interferon signaling) as well as adaptive immune signaling (e.g., BCR signaling, lymphocyte co-stimulation). Indeed, excessive immune activation is a hallmark of severe influenza pneumonia that leads to lung injury (105, 106). Conversely, reduced inflammation is associated with higher viral loads, but the clinical pathology is also less severe (107). Our results suggest nicotine primes the airspaces for increased neutrophilic inflammation but initially impairs viral clearance, which stimulates more robust activation of immune pathways not suppressed by nicotine as a compensatory mechanism.

Multiple studies have reported that e-cigarette exposure has harmful effects in the context of pathogen infections (108). Inflammation and virulence were increased by e-cigarette aerosols during respiratory infections with multiple bacterial species (55, 56, 109). Exposure to e-cigarette aerosols increased the infectivity of SARS-CoV-2 in a bronchial epithelial cell line (57, 58). E-cigarette aerosols amplified the pro-inflammatory response of distal airway epithelial cells to influenza virus (110). Mice infected with influenza virus had reduced survival and greater lung injury when exposed to e-cigarette aerosols (50). The nasal mucosa of e-cigarette users had blunted immune defense responses to live attenuated influenza virus (111). Notably, one study has reported higher lung viral load and mortality following influenza infection in mice exposed to aerosols generated by an early generation e-cigarette device (112). Importantly, these animal studies of respiratory infections investigated the whole chemical milieu in the e-cigarette aerosol (i.e., the nicotine, VG/PG and other additives in combination) and, thus, could not comment conclusively on nicotine-specific effects. In this study, we have isolated the effects of nicotine in aerosol form to downregulate critical host defense mechanisms in the lungs and impair viral clearance, upregulating immune responses and increasing lung protein permeability during influenza infection.

These findings may be especially relevant to tobacco product regulation. Over the past decade, the use of e-cigarettes has increased substantially. These devices were initially marketed as an aid for cigarette smoking cessation (14, 113), in part due to a perception that the deleterious effects of cigarettes are caused by other components of cigarette smoke. Moreover, a significant proportion of e-cigarette users, particularly youth and young adults, did not smoke cigarettes previously (114, 115). These results add to the body of evidence that nicotine is harmful even in e-cigarettes and should inform the regulation of nicotine-containing products given that the amount of nicotine in popular e-cigarette products has increased over time (12) and countries are beginning to implement limits on nicotine content (116).

Limitations of this study include the short duration of exposure of the mice considering that e-cigarette use is often a daily habit over months or years. The e-cigarette in this study was a re-fillable tank-style device, whereas the major market share is currently held by disposable pod-style devices. The advantage, though, of this tank-style device was the ability to separate effects of nicotine from the other chemical constituents, and these tank-style devices continue to be used by many daily e-cigarette users (117). Additionally, the mice were inoculated with a relatively moderate dose of influenza virus and, thus, the influence of e-cigarette exposure on mortality from infection could not be assessed. Nonetheless, these limitations indicate that further studies are necessary to understand the risks associated with e-cigarettes in a rapidly evolving commercial landscape, particularly as synthetic nicotine is introduced into the marketplace (12).

In summary, this study provides evidence for a nicotine-specific impairment of influenza viral clearance in mice exposed to e-cigarette aerosols. Additionally, aerosolized nicotine caused inflammation in the distal airspaces prior to infection. Nicotine exposure also caused a marked shift in pulmonary gene expression following influenza infection characterized by increased pro-inflammatory gene expression and downregulation of genes linked to ciliary function and fluid clearance. Aerosolized nicotine also reduced BAL MUC5AC concentration and increased lung protein permeability induced by influenza infection. These findings demonstrate nicotine has harmful effects during influenza infection in mice and provide a rationale for using viral infection susceptibility as a benchmark in guiding industry regulations and product standards for e-cigarettes.

## Data availability statement

The original contributions presented in the study are publicly available. This data can be found here: https://www.ncbi.nlm.nih.gov/bioproject/PRJNA891788.

## Ethics statement

The animal study was reviewed and approved by Institutional Animal Care and Use Committee University of California at San Francisco.

## Author contributions

CSC, JEG, and MAM conceived and designed the study. MM, AS, LFC, SC, XF, JA, and JEG performed experiments. MM, AS, LFC, SC, SAC, CRL, CSC, JEG, and MAM performed data analysis and interpretation of results. MM and AS drafted the manuscript. All authors contributed to the article and approved the submitted version.
